# Assessing the knowledge, attitude and perception of Extended Reality (XR) technology in Pakistan’s Healthcare community in an era of Artificial Intelligence

**DOI:** 10.3389/fmed.2024.1456017

**Published:** 2024-10-16

**Authors:** Zoha Khan, Talha Adil, Malik Olatunde Oduoye, Bareerah Shaukat Khan, Meher Ayyazuddin

**Affiliations:** ^1^Azad Jammu Kashmir Medical College, Muzzaffarad, Pakistan; ^2^Medical Research Circle, Bukavu, Democratic Republic of Congo; ^3^Dow Medical College, Dow University of Health Sciences, Muzaffarabad, Pakistan; ^4^CMH Lahore Medical College, Muzaffarabad, Pakistan

**Keywords:** extended reality (XR), virtual reality (VR), augmented reality (AR), mixed reality (MR), medical education, healthcare, artificial intelligence (AI)

## Abstract

**Background and objectives:**

The Extended Reality (XR) technology was established by combining elements of Virtual Reality and Augmented Reality, offering users the advantage of working in a virtual environment. The study aimed to evaluate medical professionals’ and students’ knowledge, attitudes, and practices regarding using XR technology in Pakistan’s healthcare system and identify its benefits, drawbacks, and implications for the system’s future.

**Methodology:**

A cross-sectional study was executed by circulating a self-structured online questionnaire among the Medical Community across Major Cities of Pakistan using various social media platforms as available sampling. The sample size was calculated to be 385 using RAOSOFT. Cronbach’s alpha was calculated as 0.74. The Exploratory Factor Analysis (EFA) conducted on the dataset was validated using the Kaiser-Meyer-Olkin (KMO) measure and Bartlett’s Test of Sphericity. The KMO value of 0.752 indicates adequate sampling, and Bartlett’s Test was significant (χ^2^ (435) = 2809.772, *p* < 0.001), confirming the suitability of the data for factor analysis. Statistical analysis was done using SPSS-25, and data description was done as frequency and percentage. Pearson correlation and regression analysis kept *p*-value < 0.05% significant.

**Results:**

Approximately 54.8% of 406 participants conveyed their familiarity with XR technologies. The majority of participants (83.8%) believed that using XR technology effectively enhanced medical education and patient care in Pakistan. Regarding clinical outcomes, 70.8% believed XR improved the efficiency of procedures and 52.8% agreed XR would lead to more device-dependent systems and eradicating human error (32.4%). Major barriers to XR integration included ethical and privacy issues (63.9%), lack of technological advancements in Pakistan (70%), and lack of ample knowledge and training of XR among health care professionals (45.8%). Hypothesis testing revealed a low positive but significant correlation between the use of AI-based healthcare systems and the increasing speed and accuracy of procedures (*r* = 0.342, *p* < 0.001), supporting Hypothesis 1. Similarly, a very low positive yet significant correlation was observed between the augmentation of diagnostic and surgical procedures and addressing data security and ethical issues for implementing XR (*r* = 0.298, *p* < 0.001), supporting Hypothesis 2. Lastly, a correlation between the mean Attitude (MA) score and the mean Perception (MP) score was found to be moderately positive and significant (*r* = 0.356, *p* < 0.001). Hence, the hypothesis 3 was supported.

**Conclusion:**

XR technology has the potential to enhance medical education and patient care in Pakistan, but its adoption faces significant challenges, including ethical concerns, technological gaps, and inadequate training. The study’s findings highlight the need to address these issues to maximize the benefits of XR in healthcare.

## 1 Introduction

Extended reality, which includes virtual reality, augmented reality, and mixed reality, revolutionizes people’s lives by enabling collaboration between real and virtual elements. It bridges the gap between the physical and virtual worlds (1–4). In the field of clinical care and medical education, all three extended reality technologies have the potential to bring about significant changes. Mixed reality, for instance, can enhance understanding of complex surgical procedures, anesthesia, and medications. Additionally, augmented reality can generate three-dimensional holograms from MRI scans to assist in neurological procedures and stereotactic processes (5–7).

To fully comprehend the impact of enhanced simulation and extended reality on medical education and healthcare systems, high-quality studies are needed (8, 9). Furthermore, educational reforms are necessary to equip medical students and trainees with the skills needed to tackle real-world challenges. Public health education can also benefit from extended reality, as it offers a comprehensive learning experience and promotes competency in investigating public health issues and managing infectious diseases (10–12).

To address the gaps in utilizing extended reality, a workshop was organized to evaluate operational techniques. Further research is required to explore how XR technologies can be used for the diagnosis and treatment of diseases (13, 14). An integrative review of articles focusing on extended reality in the field of medical education revealed that XR technology, including virtual reality and augmented reality, is as effective as traditional teaching methods in improving learning outcomes and patient care (15–22). While extended reality has the potential to transform various aspects of life, there is a lack of well-documented literature on how XR technology should be integrated into the healthcare system (23). Currently, there is insufficient research on the implementation of XR technologies in medical education, despite their world-changing potential (24).

Extended Reality is an unstoppable force and we should be improving our teaching methods to adapt to this change. These technologies serve clinical care by offering, psychological assessment (25–27), telehealth services (28), visualizations (29), nutritional guidance (30) and communication capabilities. Utilizing 3D capabilities, applications are designed for teaching and pre-operative planning purposes. Extended reality has showcased its effectiveness during interventional procedures by offering 3D visualizations of patient anatomy, scar visualization, and real-time catheter tracking using touch-free software control. Extended reality devices find applications in education, pre-procedural planning, and cardiac interventions (31–33). Advancements in hardware and software are propelling the expansion of XR-assisted surgery. While augmented virtuality and mixed reality remain relatively unexplored, VR utilization is increasing, particularly in surgical training and pre-operative preparation. With the impact of COVID-19 restricting physical interaction and surgical procedures becoming more complex, XR-assisted surgery is poised to assume a larger role soon (34–39).

Extended Reality has also widened the horizons of visualization capabilities, particularly with microscopic images, molecular data, and anatomical datasets. An example of such advancement is the Google AR Microscope (ARM) system, which consists of an augmented bright-field microscope, a computer, and a set of trained deep-learning algorithms. This innovative system has proven to assist pathologists in reducing the time needed to scan multiple images to detect the presence of cancer (40).

As XR technology rapidly evolves, it becomes essential to evaluate healthcare professionals’ current awareness and comprehension levels regarding these advancements. In response to this necessity, we conducted an extensive survey to measure the familiarity and knowledge of XR technology within the healthcare workforce.

Hypotheses:

1. There is a significant relationship between the “Use of AI-based healthcare systems” and the “Perceived increase in the speed and accuracy of procedures.”
2. A correlation exists between the “Perceived augmentation of diagnostic and surgical procedures” and concerns related to “Addressing data security and ethical issues for implementing XR.”
3. A correlation exists between the “level of medical education” and the “mean Attitude (MA) score and the mean Perception (MP) scores”.

## 2 Subjects and methods

### 2.1 Type of study, sample size, and sampling

A descriptive cross-sectional study was conducted by administering a structured self-administered questionnaire among the Medical Community in 2023. The STROBE (Strengthening the Reporting of Observational Studies in Epidemiology) checklist was employed in this study to enhance the quality and transparency of reporting. As this is a descriptive, observational study, the STROBE checklist provides a structured framework that ensures all critical components of the study design, data collection, analysis, and interpretation are thoroughly addressed. These elements ensure clarity, completeness, and transparency in reporting observational studies. By using this checklist, we aimed to minimize potential biases, improve the reproducibility of our findings, and ensure that our study meets the highest standards of scientific rigor and clarity. A standard sample size of 385 was selected through Raosoft for a nationwide study.

### 2.2 Study implementation

An online questionnaire to obtain wide coverage and a larger sample was distributed among the Medical Community across 9 major and most developed Cities of Pakistan using various Social Media platforms. These cities included Karachi, Lahore, Faisalabad, Rawalpindi, Islamabad, Multan Peshawar, Abbottabad and Muzzafarabad which represent all regions (provinces and territories) of Pakistan. Medical Students, House Officers, Medical Officers, Postgraduate Trainees, Professors, Specialists, and Consultants from all across Pakistan were included in the study. The duration of completing the questionnaire was 10–15 min.

### 2.3 Data collection

Knowledge, Attitude and Perception were dependent variables while socio-demographic characteristics (age, education, gender, residence and city) were independent variables. The questionnaire included sections on demographic characteristics such as gender, area of residence (rural or urban), level of medical education, major cities, and age. The questionnaire was divided into three main sections: Knowledge (9 questions), Attitude (9 questions), and Practice (11 questions). It incorporated a variety of question types, including closed-ended questions with binary responses (Yes/No/Maybe), Likert scale items (ranging from “Effective” to “Slightly Ineffective”), and multiple-choice questions offering several response options. The instrument was developed by reviewing similar studies and was modified to align with the specific context of this study. This approach ensured that the questionnaire was both comprehensive and relevant to the current research objectives.

To ensure the validity and reliability of the data collection instrument, we employed two key strategies, content validity and pilot testing. Content validity was established through expert review, where subject matter experts in XR technology, medical education, and research methodology evaluated the questionnaire to confirm that it comprehensively covered the relevant topics. Their feedback was used to refine and adjust the questionnaire, ensuring that it accurately reflected the constructs of interest. Additionally, a pilot test was conducted with a small sample of participants (20) and Cronbach’s α was calculated as 0.74. This pre-testing phase provided valuable insights into the clarity, relevance, and comprehensiveness. Based on the feedback obtained, necessary modifications were made to improve the questionnaire. These validation steps collectively ensured that the instrument was well-suited for capturing the intended data and provided reliable measures for the study. Additionally, The Exploratory Factor Analysis (EFA) conducted on the dataset was validated using the Kaiser-Meyer-Olkin (KMO) measure and Bartlett’s Test of Sphericity. The KMO value of 0.752 indicates adequate sampling, and Bartlett’s Test was significant (χ^2^(435) = 2809.772, *p* < 0.001), confirming the suitability of the data for factor analysis. Eight factors were extracted, explaining 54.13% of the total variance. Several key variables showed strong commonalities. These findings suggest a robust factor structure, supporting the validity of the constructs measured.

Data was collected through different social media platforms using a convenient sampling technique from 1st August to 30th September 2023. 406 responses were collected from the online survey (41–44). Participants filled out an online self-structured questionnaire containing 35 questions related to research problems.

### 2.4 Statistical analysis

The questionnaire was designed in a way to avoid all kinds of bias. Data was entered and transferred to Statistical Package for Social Sciences version 25 for analysis. We computed frequencies and percentages for all the categorical variables. Pearson Correlation and Regression analysis were applied to covariate the knowledge and perception with attitude variables.

### 2.5 Ethical approval

Ethical approval was obtained from the Ethical Review Committee of the Azad Jammu and Kashmir Medical College, Muzaffarabad. Moreover, consent was also taken from the participants at the start of the online questionnaire, ensuring the safety and confidentiality of the information they were given.

## 3 Results

### 3.1 Demographic information

The survey garnered a robust response, with a total of 406 participants contributing their valuable insights. Table 1 elucidates the gender distribution, age of respondents, level of education, area of residence and city of work. A predominant proportion of respondents identified as male, comprising 54.43% (*n* = 221), while 45.57% (*n* = 185) represented the female cohort. A substantial majority hailed from urban settings, constituting 88.2% (*n* = 359. The mean age emerged as 24.41 years. The lion’s share, amounting to 77.6% (*n* = 316), identified as medical students. Muzzafarabad emerged as the most prominently featured city, boasting 20.7% (*n* = 84) of the respondents.

**TABLE 1:** Demographic characteristics (*n* = 406 HCWs).

Contents				Frequency	Yes %
**Gender**					
Female				185	45.5
Male				221	54.3
**Area**					
Rural				48	11.7
Urban				359	88.2
**Level of medical education**					
House Officer				18	4.4
Medical Officer				32	7.9
Medical Student				316	77.6
Post-Graduate Trainee				16	3.9
Professor/Specialist/Consultant				25	6.1
**Major cities**					
Muzzafarabad				84	20.7%
Rawalpindi/Islamabad				77	19.0%
Karachi				63	15.5%
Others				62	15.3%
Multan				24	5.9%
Lahore				60	14.8%
Abbottabad				12	3.0%
Peshawar				19	4.7%
Faisalabad				5	1.2%
**Age**	**N**	**Minimum**	**Maximum**	**Mean**	**Std. Deviation**
Age of respondents	406	17	70	24.41	8.109

### 3.2 Knowledge

The data shows that among the total participants, 67.7% were knowledgeable about Artificial Intelligence. About 51.2% expressed strong support for the integration of extended reality (XR) into the healthcare system. Improved patient education, therapy and management (47%) and simplified hospital navigation (44.6%) were identified as the foremost healthcare domains for utilizing extended reality (XR). Additionally, 42.61% believed it contributed to a decrease in risk during medical procedures, while 38.67% saw its value in providing limitless medical practice opportunities and personalized learning experiences for medical students. Table 2 sheds light on the mentioned knowledge information.

**TABLE 2:** Knowledge of HCWs regarding XR technology (*n* = 406).

Variable		Percent
Familiarity of Healthcare professionals with XR Technology in healthcare	Heard of it but not sure about the details	54.8
	Not familiar at all	35.3
	Very familiar	9.8
How would you rate the effectiveness of XR technology in enhancing medical education and patient care?	Effective	51.1
	Ineffective	2.2
	Neutral	20.1
	Slightly effective	21.4
	Slightly ineffective	4.9
Where have you heard about Artificial Intelligence?	Conversations with family and friends	3.9
	Educational Institute	8.8
	News articles or Websites	9.8
	Social Media Platforms (e.g., Facebook, Instagram, Twitter etc.)	67.6
	Tech Conferences or events	4.7
	TV shows or Documentaries	5.2
Healthcare domains for utilizing extended reality (XR)	Improved patient education, XR assistive therapy and management	47.0%
	Augmented diagnosis and surgeries	18.0%
	Simplified hospital navigation	44.6%
	Medical education and research	15.0%
	Pharma marketing and advertising	0.0%
Benefits of integrating XR in healthcare.	Capable of telemedicine practice	21.7%
	Education and improved care for patients	51.0%
	Collaborative environments	22.4%
	Unlimited practice and personalized learning for students	38.7%
	Lower risk and higher understanding	42.6%
Is there any ethical limitation regarding XR technology in healthcare system?	No	24.0%
	No idea	35.1%
	Yes	40.8%
Can XR be used to improve the healthcare system, for advancements in medical field.	May be	17.6%
	No	2.7%
	Yes	79.6%
Do you know any organization or hospital using XR technology in Pakistan?	No	72.2%
	Yes	27.8%
Have you personally used any XR technology in medical education purpose?	No	84.0%
	Yes	16.0%

### 3.3 Attitude

48% viewed the inclusion of extended reality (XR) in Pakistan’s healthcare system as a significant advancement. 80.6% of respondents believe XR technology is as effective as traditional techniques, followed by 78.6% expressing confidence in its potential to enhance medical education. Moreover, 70.8% anticipate that incorporating AI into the system will lead to faster and more accurate medical procedures. Further details on participants’ attitudes towards XR technology in healthcare are presented in Table 3 below.

**TABLE 3:** Attitude of HCWs regarding XR Technology (*n* = 406).

Variable		Percent
Do you believe that XR technology can improve medical education and training?	May be	17.2%
	No	4.2%
	Yes	78.6%
Do you consider XR technology to be as effective as traditional techniques for the healthcare system?	May be	0.2%
	No	19.2%
	Yes	80.6%
Do you think procedures will be faster and more accurate by incorporating AI into the systems?	May be	18.9%
	No	10.3%
	Yes	70.8%
Do you think revolutionizing the systems with XR technology would be more device-dependent rather than operator-dependent?	May be	26.5%
	No	20.6%
	Yes	52.8%
Are you concerned about ethical and privacy issues XR technology would raise?	May be	0.2%
	No	35.9%
	Yes	63.9%
Do you think adopting XR technologies reduces the transmission of infectious diseases?	May be	23.8%
	No	19.7%
	Yes	56.5%
Do you think XR technology will affect/limit doctor-patient interaction would lead to skepticism and legal issues?	May be	29.5%
	No	22.3%
	Yes	48.2%
Do you believe that it would promote a remote working environment in healthcare systems?	May be	19.9%
	No	11.7%
	Yes	68.3%
What are your views about incorporating XR technology in your medical practice or studies?	Insignificant	0.7%
	Neutral	16.7%
	Not much	4.2%
	Significant	48.4%
	Somewhat possible	30.0%

### 3.4 Perception

Some perception questions with responses are shown in Table 4. A (61.9%) express the importance of integrating AI-based healthcare systems post-COVID-19. Notably, 83.8% anticipate XR technology as the future of healthcare in resource-limited developing countries. Furthermore, 70.5% consider XR as cost-effective, supporting remote health services, while 70% believe it can minimize delays in network communication. Conversely, a minority (32.4% and 29.2%) holds concerns about XR potentially increasing medical errors and replacing doctors’ in future healthcare systems. A significant majority of 83.8% anticipate XR technology as the future of healthcare in resource-limited developing countries. Participants identified high costs (67.73%) as the most potential barrier to the integration of Extended Reality into Pakistan’s healthcare system.

**TABLE 4:** Perception of HCWs related XR Technology (*n* = 406).

Variable		Percent
Do you think adopting AI based healthcare system is integral after COVID-19	May be	23.5%
	No	14.5%
	Yes	61.9%
Do you want to see XR technology as future of healthcare in developing countries like Pakistan with limited healthcare resources?	May be	0.2%
	No	16%
	Yes	83.8%
Do you think XR relatively increases chances of medical error?	May be	32.5%
	No	35.1%
	Yes	32.4%
Do you think the delay in network communication (3G, 4G) can be a limitation in adopting this system?	May be	20.3%
	No	9.6%
	Yes	70%
Do you think, XR technology would be cost-effective and promote remote health services?	May be	0.2%
	No	29.2%
	Yes	70.5%
Do you think adopting new systems of healthcare would replace doctors in future?	May be	21.8%
	No	48.9%
	Yes	29.2%
How do you perceive the adaptation of XR technology in healthcare?	Cutting-edge and transformative	20.1%
	Neutral - neither positive nor negative	24.6%
	Not convinced of its benefits at all	3.7%
	Promising but needs further development	42.0%
	Skeptical about its practicality	9.6%
Barriers perceived	High costs of XR equipment	67.7%
	Limited access to XR technology	47.8%
	Lack of evidence-based research supporting XR in healthcare	38.9%
	Concerns about patient acceptance and safety	40.6%
	Lack of knowledge/training on XR usage	45.8%
	Uncertainty of its benefits over traditional methods	0.0%
Significantly benefited areas within the healthcare sector.	Medicine (neurology, cardiology, pediatrics, dermatology, ophthalmology etc.)	45.8%
	Basic Sciences and Research	32.5%
	Gynecology and Obstetrics	18.2%
	Surgery (neurosurgery, plastic surgery, and orthopedics etc.)	65.3%
	Radiology	48.5%
Essential factors for successful XR implementation in healthcare.	Cost-Effectiveness	61.8%
	Remote Patient Monitoring and Telemedicine	27.3%
	Healthcare Professional Training and Infrastructure	56.9%
	Patient Acceptance and Education	44.1%
	Data Security and Ethical Considerations	36.5%
How likely are you to recommend the use of XR technology to your colleagues or institution for healthcare applications?	Likely	37.1%
	Neutral	29.5%
	Unlikely	4.2%
	Very likely	27.0%
	Very unlikely	2.2%

### 3.5 Correlation analysis

Hypothesis 1: Pearson product correlation between the “Use of AI-based healthcare system” and “Increasing speed and accuracy of procedures” was found low positive but significant (*r* = 0.342, *p* < 0.001). Hence, hypothesis 1 was supported (Table 5).

**TABLE 5:** Correlational analysis.

	Level of Medical Education	Enhancing medical education and patient care	Increasing speed and accuracy of procedures using AI	Use of AI based healthcare system after COVID-19	XR will replace doctors in future	Recommending use of XR technology
Level of Medical Education	1					
Enhancing medical education and patient care	−0.128**	1				
Increasing speed and accuracy of procedures using AI	0.027	−0.220**	1			
Use of AI-based healthcare system after COVID-19	0.032	−0.100*	0.342**	1		
XR will replace doctors in future	0.033	−0.055	0.142**	0.190**	1	
Recommending the use of XR technology	0.137**	−0.064	−0.001	0.124*	0.180**	1

“*” means correlation is significant at 0.05,

“**” means correlation is significant at 0.01.

Hypothesis 2: Pearson product correlation between “Augmentation of diagnostic and surgical procedures” and “Addressing data security and ethical issues for implementing XR” was found to be very low positive but significant (*r* = 0.298, *p* < 0.001). Hence, hypothesis 2 was supported (Table 6).

**TABLE 6:** Correlational analysis.

	XR improves patient education, assisted therapy and management	XR augments diagnosis and surgeries	XR equipment has a high cost	XR has uncertain benefits over traditional methods	Addressing data security and ethical issues for implementing XR
XR improves patient education, assisted therapy and management	1				
XR augments diagnosis and surgeries	0.163**	1			
XR equipment has a high cost	0.165**	0.227**	1		
XR has uncertain benefits over traditional methods	.^b^	.^b^	.^b^	.^b^	
Addressing data security and ethical issues for implementing XR	0.199**	0.298**	0.205**	.^b^	1

“**” means correlation is significant at 0.01. Superscripted values were significant.

**FIGURE 1:**
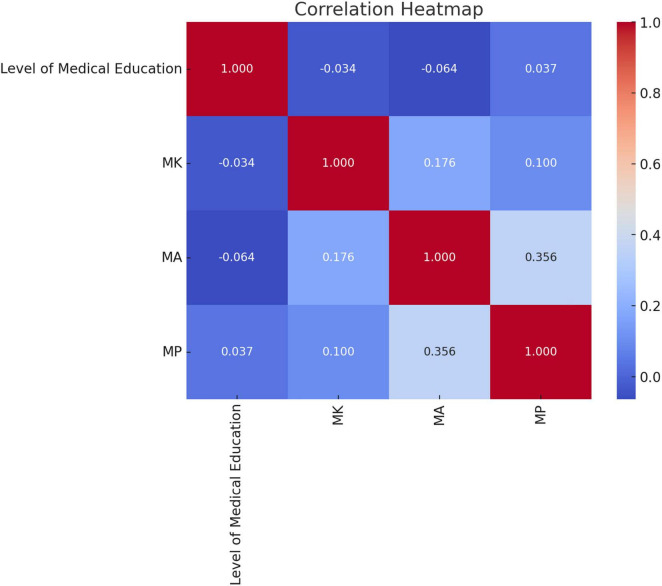
Heatmap for correlation.

Hypothesis 3: Pearson product correlation between the mean Attitude (MA) score and the mean Perception (MP) score was found to be moderately positive and significant (*r* = 0.356, *p* < 0.001). Hence, the hypothesis 3 was supported (Figure 1).

### 3.6 Regression analysis

The hypothesis tests if knowledge and perception factors have a significant impact on the attitude of healthcare professionals. The dependent variable recommendation and implication of XR technology in institutions were regressed on predicting variables high cost of XR technology, adopting new systems of healthcare would replace doctors in future, the scope of XR technology in enhancing medical curriculum, patient care, data security and ethical considerations, procedures will be revolutionized by incorporating AI into the systems, adopting AI-based healthcare system is integral after COVID-19 to test the hypothesis. The above-mentioned variables significantly predicted that the overall model is statistically significant [F (6, 399) = 3.390, *p* = 0.003]. This suggests that at least one of the predictors has a significant effect on the likelihood of recommending XR technology in healthcare.

Hypothesis H1 is the relationship between the belief that new healthcare systems would replace doctors in the future and the likelihood of recommending XR technology. The adoption of new systems of healthcare that would replace health professionals in future can play a significant role in implementing XR (b = 0.180, *p* = 0.0001). The coefficient is statistically significant (*p* = 0.0001), indicating a positive and significant impact on the likelihood of recommending XR technology.

Hypothesis H2 is that adopting AI-based healthcare systems is integral after COVID-19 and the likelihood of recommending XR technology. The adoption of AI-based healthcare systems after COVID-19 is statistically important in implementing the XR technology (b = 0.124, *p* = 0.013). The coefficient is statistically significant (*p* = 0.013), suggesting a potential positive influence on the likelihood of recommendation.

Hypothesis H3 is the belief that procedures will be faster and more accurate with AI incorporation and the likelihood of recommending XR technology. Lastly, healthcare procedures will be faster and more accurate by incorporating AI into the systems plays a significant role in implementing XR (b = −0.146, *p* = 0.088). The coefficient is marginally significant (*p* = 0.088), indicating a possible but not fully established impact on the likelihood of recommending XR technology (Table 7 shows the summary of the findings).

**TABLE 7:** Regression analysis.

Hypothesis	Beta coefficient	*R* ^2^	*F*	*t*-value	*p*-value	Hypotheses supported
H1	0.180	0.033	13.611	**3.689**	0.0001	Yes
H2	0.124	0.015	6.293	**2.509**	0.013	Yes
H3	−0.146	0.049	6	−**1.710**	0.088	Marginally yes

Bold values indicate the t-value, measuring how many standard errors the coefficient is away from zero.

## 4 Discussion

The integration of Extended Reality (XR) technology in healthcare is becoming increasingly significant, especially within the context of artificial intelligence (AI). In this study, 54.43% of the participants were male, while 45.57% were female. However, in Jordan, it was found that 63.8% were female and 36.2% were male with a median age of 21 (range 21–22) as compared to ours mean age emerged as 24.41 years, accompanied by a standard deviation of 8.109 and a range spanning from 17 to 70 years. Our study’s participant age range is notably broader, which could imply varying levels of familiarity and comfort with emerging technologies across different age groups. This statistical portrait paints a picture of a relatively youthful participant pool (45). The occupational affiliations of the respondents in our study yielded an intriguing panorama. The majority amounting to 77.6% (*n* = 316), identified as medical students as compared to the above-mentioned study having medicine/dentistry (47.6%) suggests that the integration and acceptance of XR technologies might differ based on the specific medical training and exposure levels. These comparative insights underscore the necessity of tailoring XR technology integration strategies to the unique demographic and occupational characteristics of target populations. As the healthcare sector increasingly adopts AI and XR technologies, understanding these nuances will be critical in ensuring effective implementation and maximizing the potential benefits of these innovations.

Our results uncovered that a substantial majority 54.9% were cognizant of XR technology, but their grasp of its practical applications in healthcare was restricted as shown in Table 2. This gap in knowledge highlights the necessity for more focused educational initiatives to bridge the divide between general awareness and practical application. Comparatively another study in Middle East showed that a significant proportion of participants claimed not to understand the basic computational principles of AI (365, 41.7%). Merely a small subset of 10% demonstrated a comprehensive understanding of the potential advantages and implementation approaches of XR technology across diverse healthcare settings, while 34.4% of respondents exhibited no awareness at all underscoring the importance of increasing exposure and education around these innovations. This is concerning, as it points to a need for more targeted educational efforts to ensure healthcare professionals can effectively harness these technologies. 67.7% were knowledgeable about Artificial Intelligence as they predominantly gather information about AI through social media platforms like Facebook, Instagram, and Twitter, followed by sources such as news articles, videos, TV shows, documentaries, tech conferences or events, and discussions with family and friends as compared to the above mentioned study as familiarity with AI nomenclature, the majority of participants were familiar with algorithms (461 and 52.7%). These results underscore a varied and comprehensive approach to acquiring AI knowledge, underscoring the significant influence of social media as a primary information source and its potential as a powerful tool for spreading awareness and education on technological advancements. A significant majority of respondents (61.9%) express the importance of integrating AI-based healthcare systems post-COVID-19 reflecting a growing recognition of the role AI can play in enhancing healthcare delivery as compared to the above mentioned study AI application impact during COVID-19 (score 2.5 ± 2.24 out of 7). These findings underscore the urgency of developing strategies to better educate and prepare healthcare professionals for the integration of AI and XR technologies in the post-pandemic world (46).

In addition to our findings, where 51% of participants identified the enhancement of medical education as the primary advantage of extended reality (XR) technology, there is growing evidence from other regions that supports the integration of artificial intelligence (AI) in medical training. A study conducted at Ain Shams University revealed a strong consensus among medical students regarding their familiarity with and the utilization of AI in their education. This reflects a broader recognition of the transformative potential AI holds in shaping the future of medical practice, aligning with the increasing global demand for AI literacy among healthcare professionals (47). Another study in Jordan showed the demand for AI/ML education in medical school or residency; the majority of the respondents 62.8% (*n* = 565) strongly agreed on the need. Moreover, a study in Jordan further underscores this trend, with 62.8% of respondents strongly agreeing on the necessity of incorporating AI and machine learning (ML) education into medical school curricula or residency programs. This overwhelming support highlights a critical shift in educational priorities, where future healthcare professionals are expected to be well-versed in AI/ML technologies, not merely as an adjunct but as an integral part of their training. The alignment of these findings across different geographical and educational contexts reinforces the urgent need for a paradigm shift in medical education, where both XR and AI are seen not just as enhancements but as essential components of a modern, comprehensive medical curriculum (48). Another study highlights the importance of conducting OSCE using an augmented reality simulator (49). Another study showed that incorporating image overlay projection, a form of augmented reality (AR), into surgical procedures has shown promise in enhancing the intuitiveness of computer-aided surgery. This technology allows surgeons to view underlying anatomical structures directly on the patient’s surface, eliminating the need to divert their sight between the patient and a display screen. It also aids in the 3-D understanding of anatomical structures and the identification of critical areas during surgery (50). These insights suggest a growing recognition among medical students and educators alike of the need to adapt to technological advancements. The convergence of XR and AI in medical education offers a unique opportunity to enrich learning experiences, improve patient outcomes, and prepare the next generation of healthcare providers for the challenges and opportunities presented by these cutting-edge technologies.

This study revealed that a majority (51.2%) of participants expressed strong support for the integration of extended reality (XR) into the healthcare system, with 48% viewing its inclusion in Pakistan’s healthcare as a significant advancement. These findings underscore the growing recognition of XR’s potential to revolutionize healthcare delivery. However, realizing this potential requires further research, development, and strategic implementation to ensure that XR technologies are effectively integrated and optimized for clinical use. The enthusiasm for XR in healthcare parallels findings from a study in Saudi Arabia, where more than half of the students (57.3%) recognized that AI could significantly enhance the capabilities of healthcare professionals. This alignment between XR and AI highlights a broader trend of technological acceptance within the healthcare community, where both innovations are seen as pivotal in advancing medical practice (51). In our study, participants identified improved patient education, therapy and management (47%), and simplified hospital navigation (44.6%) as the primary healthcare domains where XR could make a substantial impact. This is consistent with the Saudi Arabian study, where 56.7% of students agreed that AI facilitates patient education, suggesting a shared belief across different regions in the value of technology-driven patient engagement. These insights reflect a growing consensus on the transformative potential of XR and AI in healthcare. As these technologies continue to evolve, they are likely to play increasingly critical roles in enhancing patient care, streamlining healthcare processes, and improving overall healthcare outcomes. However, to fully harness these benefits, concerted efforts in education, training, and infrastructure development will be essential (51).

This study highlights several key applications of Extended Reality (XR) in healthcare, particularly in augmented reality (AR)-assisted diagnosis and surgeries (39%), as well as in medical education and research (36%). These findings reflect the growing interest in utilizing XR technologies to enhance various aspects of healthcare, offering immersive and interactive experiences that have the potential to revolutionize patient care, clinical practice, and medical training. Additionally, a study highlights that advanced artificial intelligence (AI) systems are emerging as pivotal players in the transformation of orthopedic surgery and post-operative rehabilitation. One notable feature of these AI systems is their ability to detect early deviations or delays in a patient’s rehabilitation process. An example of such technology is WalkAI, an AI component integrated into the myMobility app within the ZBEdge ecosystem. WalkAI exemplifies how technology is advancing the quality of care provided to patients undergoing knee prosthetic surgeries, by offering personalized insights and improving overall outcomes in their recovery journey (52). In addition to the advancements in XR and AI technologies mentioned earlier, AI-based tools are increasingly being utilized throughout the entire process of anterior cruciate ligament (ACL) reconstruction. A recent review highlights the current clinical applications and prospects of AI in this field, focusing on its role in preoperative management, intraoperative assistance, and postoperative care. Before surgery, AI tools are being used for risk prediction and diagnostics, helping to identify patients who may benefit most from ACL reconstruction. During the surgery itself, AI-driven navigation systems aid surgeons by identifying complex anatomic landmarks, thereby improving the precision and outcomes of the procedure. Postoperatively, AI is playing a crucial role in patient care and rehabilitation, particularly in monitoring progress and predicting potential complications. Moreover, AI tools are becoming integral to educational and training settings, where they are used to simulate surgical procedures and enhance the training of orthopedic surgeons. The growing interest among orthopedic surgeons in AI, particularly in applications related to ACL injuries, is evident from the increasing number of studies dedicated to this area. This surge in research suggests that AI will have a significant future impact on the clinical management of ACL tears, with orthopedic surgeons paying close attention to these technological advancements. Integrating these AI-based tools into standard practice could lead to more personalized and effective treatment strategies, ultimately improving patient outcomes in orthopedic surgery (53). Another study highlights a notable feature of AI-based tools in orthopedic surgery is their high accuracy and precision, particularly when comparing implant positioning with preoperative targeted angles, with errors typically within ≤ 2 mm and/or ≤ 2°. Despite these promising results, which demonstrate significant improvements in accuracy and precision, this technology is still far from being widely adopted in daily clinical practice (54).

The transformative potential of XR is further underscored by a study conducted in the Middle East, focusing on pharmacy students’ attitudes toward AI in pharmacy and pharmacy practice. The study revealed that a significant majority believed AI would improve and revolutionize clinical pharmacy practice (67.8%) and other general pharmacy sciences (71.3%). This shared belief in the power of emerging technologies, whether XR or AI, to revolutionize healthcare practice suggests a broader trend towards the acceptance and integration of these innovations across various healthcare domains. XR, with its capability to provide immersive and interactive experiences, holds particular promise in pharma marketing, patient therapy, and pharmaceutical education. By enabling more engaging and effective ways to deliver information and training, XR can significantly enhance the efficacy of healthcare delivery, making it a valuable tool for both healthcare professionals and pharmaceutical entities. These insights highlight the necessity for continued research and development in XR and AI, particularly in understanding how these technologies can be best applied to meet the evolving needs of the healthcare sector. As these technologies become more widely adopted, they have the potential to not only enhance clinical outcomes but also to redefine the very framework of modern healthcare practices (46).

Additionally, our study uncovered that 42.61% of participants recognized the potential of extended reality (XR) to reduce risks during medical procedures, while 38.67% appreciated its value in offering limitless medical practice opportunities and personalized learning experiences for medical students. These findings underscore the growing optimism surrounding XR’s ability to enhance the safety and quality of healthcare delivery, particularly in educational and procedural contexts. This sentiment is echoed in a study from Saudi Arabia, where a significant majority (69.7%) of participants strongly agreed that AI is useful in clinical decision-making, such as in the justification of examinations. The recognition of AI’s role in enhancing clinical accuracy and decision-making aligns with our findings on XR, suggesting a shared belief in the power of emerging technologies to support and improve healthcare outcomes (55).

However, it’s important to note that not all views are entirely optimistic. In our study, a minority of participants (32.4% and 29.2%) expressed concerns about the potential for XR to increase medical errors and even replace doctors in the future. This highlights a critical area of apprehension that must be addressed through careful implementation, rigorous testing, and ongoing education to ensure that XR technologies complement rather than compromise healthcare practice. In contrast, a study in Jordan revealed that 28.2% of participants believed clinical AI could surpass the accuracy of physicians, reflecting a growing trust in the capabilities of AI. These mixed sentiments emphasize the need for a balanced approach to integrating these technologies, ensuring that their benefits are maximized while addressing potential risks and ethical considerations (45).

This study’s findings on XR are juxtaposed against broader concerns about the role of AI in healthcare and education. For instance, a study in Jordan revealed that 34.1% of participants believed that human teachers would be replaced in the foreseeable future, highlighting a significant apprehension about the impact of AI on traditional roles (45). This concern is mirrored in another study from the Middle East, where 58.9% of participants viewed AI as a partner that would assist them in performing their duties effectively, while a substantial 36.4% saw it as a competitor that could potentially take over their jobs (46). These mixed perceptions extend to the medical field as well. A study involving medical students in Jordan found that a majority (69.6%) strongly disagreed with the notion that AI will eventually replace human doctors, underscoring a strong belief in the irreplaceable value of human judgment and expertise in clinical practice. However, more than half of the respondents (60.6%) agreed that having advanced personal AI/ML knowledge would enhance their professional performance, reflecting a recognition of the benefits of AI as a tool for augmenting, rather than replacing, human capabilities (48). These findings highlight the dual nature of AI and XR technologies in healthcare and education: while they hold the potential to significantly enhance and transform these fields, they also bring with them concerns about displacement and the need for careful consideration of their integration. As we move forward, it is essential to strike a balance that maximizes the benefits of these technologies while addressing the ethical and professional challenges they present.

This study identified several key barriers to the integration of Extended Reality (XR) into Pakistan’s healthcare system. The most significant challenges cited by participants were high costs (67.73%), limited access (47.78%), lack of knowledge (45.81%), patient acceptance issues (40.64%), and insufficient research (38.92%). These barriers underscore the complexities involved in adopting innovative technologies within resource-constrained environments, where financial, educational, and infrastructural limitations can hinder progress. These findings resonate with those from a study in Jordan, where 58.9% of participants recognized similar barriers to the application of AI in medicine. The most frequently reported obstacles in the Jordanian context were a lack of knowledge and expertise, followed by time constraints due to the educational burden, and insufficient access to technical equipment (52.8%, 43.1%, and 42.4% respectively) (45). The parallel between these studies highlights the universal challenges faced by developing nations in integrating advanced technologies into their healthcare systems. Whether dealing with XR or AI, the barriers of cost, access, and education remain pervasive. Addressing these issues will require targeted strategies, including investment in infrastructure, comprehensive training programs, and efforts to increase awareness and acceptance among both healthcare providers and patients. Ultimately, overcoming these barriers is crucial for unlocking the full potential of XR and AI in transforming healthcare, particularly in settings where resources are limited. Strategic planning and international collaboration may provide the necessary support to bridge these gaps and pave the way for more widespread and effective implementation of these technologies.

In this present study, data security and ethical considerations were highlighted by 36.4% of participants, while 27.3% emphasized the importance of remote telemedicine as a crucial aspect of healthcare delivery. These concerns reflect the growing awareness of the ethical implications and security challenges associated with the integration of advanced technologies like Extended Reality (XR) in healthcare. Comparatively, a study conducted in Jordan revealed that ethical and privacy concerns were the least frequently reported barrier, with only 34.2% of participants identifying them as significant. This difference may suggest varying levels of awareness or prioritization of ethical issues in different regions. However, the importance of addressing these concerns cannot be understated, especially as healthcare systems increasingly rely on digital and immersive technologies. Further supporting the need for ethical vigilance, a study in Saudi Arabia found that a substantial 70.1% of participants advocated for training programs aimed at preventing and resolving ethical issues related to artificial intelligence (AI) applications. This highlights a widespread recognition of the potential ethical challenges posed by AI and XR technologies, and the necessity for proactive measures to ensure their responsible use. These findings underscore the critical role of ethical management in the successful adoption of XR technology. As healthcare systems evolve, ethical considerations, data security, and privacy protections must be integrated into the design and implementation of these technologies. By addressing these issues head-on, healthcare providers can better manage costs, mitigate risks, and ensure that XR and AI technologies are used to enhance, rather than compromise, healthcare delivery.

A descriptive study conducted on older adults revealed a generally positive attitude toward the use of Virtual Reality (VR) technology, with participants perceiving it as both useful and enjoyable. These findings suggest that older adults could become a key demographic for the development of VR applications aimed at supporting active aging. The positive reception of VR among this group highlights its potential for enhancing quality of life and promoting engagement in various activities as they age (56). However, contrasting perspectives were uncovered in a separate study involving in-depth interviews with older individuals. In this research, participants expressed reservations and discomfort with VR technology, indicating that they did not view it as essential in their daily lives. This dichotomy underscores the broader challenge of societal acceptance of new technologies, particularly among older populations, where adoption may be slower due to unfamiliarity or perceived irrelevance. Despite these reservations, the study suggests that VR may find greater acceptance and application in professional domains, such as medical treatment and e-learning, where its benefits are more immediately tangible and practical (57). Moreover, the integration of Extended Reality (XR) in surgical processes has been explored in various studies, with promising results. XR-integrated platforms have been shown to provide valuable support for operating room (OR) teams, particularly in monitoring patient health during complex surgical procedures. These findings underscore the potential of XR to enhance surgical precision, improve patient outcomes, and reduce the cognitive load on surgeons and OR staff (58–61). These studies highlight the nuanced and context-dependent nature of technology acceptance. While VR and XR hold significant promise in both consumer and professional settings, their successful adoption will require addressing user-specific concerns and demonstrating clear, practical benefits. As the technology continues to evolve, targeted strategies to improve familiarity and comfort with VR and XR among older adults and healthcare professionals will be essential to fully realize their potential.

An analytical study investigating the use of Extended Reality (XR) for medical training and clinical support during space missions revealed its critical importance in such high-stakes environments. The study demonstrated that XR technology is invaluable for diagnosing conditions and planning treatment courses during emergency situations in space. This finding highlights the potential of XR to provide essential medical support in remote and resource-limited settings, where traditional medical resources are not readily accessible (62, 63). In a related study conducted at Seoul National University Hospital, researchers assessed the application of XR technology for the diagnosis, grading, staging, localization, and surgical removal of lung cancers. The results were highly encouraging, indicating that XR can significantly enhance the accuracy and effectiveness of these complex medical procedures. The study’s findings underscore the transformative potential of XR in oncology, particularly in improving surgical outcomes and precision in cancer treatment (64). These studies emphasize the broad applicability of XR technology across diverse medical contexts—from the unique challenges of space medicine to the precision demands of oncological surgery. As XR continues to develop, its integration into medical practice could lead to significant advancements in both emergency care and surgical procedures, ultimately improving patient outcomes in a wide range of scenarios.

Considering these findings, the training of medical students emerges as a primary target for the integration of XR technology in the future. Our study also highlights the critical role of evidence-based research in shaping the attitudes of young medical students toward adopting XR in healthcare. Addressing the barriers to XR adoption, such as limited exposure and understanding, and promoting further research in this field, will be crucial in fostering the successful integration of XR technology into medical education and healthcare practices. By equipping future healthcare professionals with the necessary skills and knowledge, XR can be effectively leveraged to enhance both medical training and patient care (65).

A similar perspective can be applied to the fields of telemedicine and education, where XR technology offers the potential to revolutionize learning experiences. Students can access a wider range of educational opportunities globally, gaining exposure to different specialties without the constraints of time and cost. The positive feedback from medical students in major cities across Pakistan underscores the readiness for integrating XR into the country’s outdated medical education system. Technological advancements, such as XR, are reshaping the landscape of medical education and training in Pakistan. As this technology continues to evolve, it becomes increasingly important for healthcare training curricula to adapt, preparing future physicians to harness these innovations. By doing so, we can achieve greater efficacy and improved outcomes in both education and patient care, ensuring that our medical professionals are equipped to meet the demands of modern healthcare (66–69).

### 4.1 Conclusion

The findings of this study suggest that there is a consensus on the knowledge and perception of XR among healthcare professionals. To improve the quality of health education, enhance patients’ care, detect and diagnose disease before it is too late or keep a record of patients all over the country, incorporating extended reality can be a game-changing factor.

The results shows that individuals with a background in medical education and research were more likely to opt for XR technology in healthcare. These findings suggest that there is a meaningful relationship between medical education and research and the adoption of XR technology in healthcare, highlighting the importance of educational and research factors in the uptake of this innovative technology. Moreover, results are evidence of the fact that Surgery, Radiology, and Medicine were thought of as fields that significantly benefited the healthcare sector. This indicates a consensus among respondents regarding the perceived positive impact of Extended Reality technology in these specific medical fields, suggesting potential advancements and improvements in surgical, radiological, and medical practices.

In conclusion, future studies should evaluate differences between the experiences of healthcare professionals with and without an extended reality, for a better understanding of educational and surgical procedures should be sought. Featuring the use of this vital preliminary data, we will then be able to further refine and modify our survey. For instance, we might use several forms with varied question types to assess precisely how healthcare personnel who have previous knowledge of XR and those who do not see it differently. Longitudinal studies might shed light on how XR is being used in medical research and teaching. Finally, qualitative research techniques like in-depth interviews may help us comprehend the experiences of healthcare professionals using XR.

### 4.2 Limitations

Despite following a thorough approach, our study has several limitations. Bias is an inevitable component of the cross-sectional survey approach. The responses provided by research participants are the only factors that determine the study’s outcomes. Respondent bias may exist because the researchers cannot interview respondents to clarify their views or justify their responses to specific questions. Uncontrolled variables that cause problems include individual predispositions towards technology, variability in the training curricula that are evolving, and varying exposure levels to XR technology among Pakistani institutions. Despite our study presenting an insight into the attitudes, understanding, and perceptions of XR among healthcare professionals at certain institutions across the nation, it is important to exercise caution when extending these findings to the larger medical community.

### 4.3 Recommendations

According to the study “Virtual Horizons: Assessing the Knowledge, Attitude and Perception of XR Technology in Pakistani Healthcare Community in an Era of Artificial Intelligence,” it is advised that healthcare professionals undergo specialized training to improve their ability to use XR technology to its full potential. Educating healthcare workers about the advantages of XR technology through awareness campaigns and workshops can help to promote its acceptance and incorporation (70–72).

To protect patient privacy and trust, it is essential to establish explicit ethical principles and data security protocols for the use of XR technology in healthcare settings. Promoting cooperative research endeavors among medical establishments, technological specialists, and legislators might also investigate the possibilities of XR technology in enhancing medical education and patient treatment (73, 74).

To revolutionize Healthcare through Extended Reality, detailed research work is required. It is necessary to study the positive and negative effects of extended reality in detail along with patients’ and doctors’ opinions to assess its feasibility and implementation in the years to come (75–77). Factors such as cost effectiveness, technical training and social acceptance should also be considered while undertaking such long-term projects and plans (44).

## 5 Abbreviations

(1) Medical Community: This includes Medical Students, House Officers, Medical Officers, Postgraduate Trainees, Specialists, Consultants and Professors.
(2) Extended Reality: An umbrella term encapsulating Augmented Reality (AR), Virtual Reality (VR), Mixed Reality (MR), and everything in between.
(3) Augmented Reality: It is a technology that overlays digital elements, such as images, text, or animations, onto the real world.
(4) Virtual Reality: It is a technology that creates a completely computer-generated environment. It immerses users in a virtual world, blocking out the real surroundings.
(5) Major Cities: Karachi, Lahore, Faisalabad, Rawalpindi, Islamabad, Multan Peshawar, Abbottabad and Muzaffarabad. The “OTHERS” option was also given if the Medical Community from other cities wanted to participate.

## Strengths and limitations

1. Conducted an analytical cross-sectional study in 2023 among 406 medical professionals in Pakistan using a self-structured online questionnaire.
2. Surveyed participants from nine major cities via social media platforms, lasting from August 1 to September 30, 2023, with 406 responses collected.
3. Analyzed data using SPSS version 25, focusing on knowledge, attitude, and perception correlations through Pearson Correlation and Regression analyses.
4. Ethical approval obtained from the Ethical Review Committee of Azad Jammu and Kashmir Medical College, ensuring participant confidentiality and safety.
5. Study limitations included inherent biases of cross-sectional surveys and uncontrolled variables influencing respondent perspectives on XR technology in healthcare.

## Data Availability

The datasets presented in this study can be found in online repositories. The names of the repository/repositories and accession number(s) can be found below: khan, Zoha (2024), “Virtual Horizons: Assessing the Knowledge, Attitude and Perception of XR Technology in Pakistani Healthcare Community in an Era of Artificial Intelligence”, Mendeley Data, V1, doi: 10.17632/wvknnx4gry.1.
